# Reversible inhibition of sperm under guidance as an intratubular and reversible contraception in female rats: An experimental study

**DOI:** 10.18502/ijrm.v19i1.8179

**Published:** 2021-01-25

**Authors:** Abdul Salam Ansari, Kiran Sevliya, Ayesha Badar, Nirmal Kumar Lohiya

**Affiliations:** Department of Zoology, Center for Advanced Studies, University of Rajasthan, Jaipur, Rajasthan, India.

**Keywords:** Contraception, Fallopian tube, Tubal occlusion, RISUGⓇ, Reversible

## Abstract

**Background:**

Reversible inhibition of sperm under guidance (“RISUGⓇ”) is a promising intravasal male contraceptive.

**Objective:**

An exploratory study was conducted with a concept of non-invasive, transcervical, single-intervention and reversible contraception using RISUGⓇ in females.

**Materials and Methods:**

In this experimental study, 60 adult Wistar albino female rats weighing 150-155 g, 3-4 months old were divided into four groups: group I: sham-operated control; group II: tubal occlusion with RISUG for 90 days; group III: tubal occlusion with RISUGⓇ for 90 days and reversal with dimethyl sulphoxide and group IV: tubal occlusion with RISUGⓇ for 90 days and reversal with 5% NaHCO. Animals were subjected to bilateral fallopian tube occlusion with RISUGⓇ and reversal with DMSO and NaHCO${}_{3}$. The estrous cycle, fertility and histology of fallopian tube were evaluated.

**Results:**

Group I showed 100% fertility during all mating schedules. Animals of experimental groups indicated positive mating, but 0% fertility was evident following 30, 60, and 90 days of tubal occlusion. However, after reversal, fertility steadily increased to normalcy in groups III (50% at 45 days, 80% at 105 days, 100% at 150 and 195 days) and IV (70% at 45 and 105 days, 100% at 150 and 195 days) animals. Group II illustrated disorganized inner cell linings and eosinated RISUGⓇimplant-filled lumen. Reversal groups (III and IV) revealed complete restoration of cellular histo-architecture. Regular estrous cycle was noticed in all experimental groups.

**Conclusion:**

RISUGⓇ is suitable for single intervention, intratubular, reversible contraception in female rats.

## 1. Introduction

Family planning issues are often a significant cause of concern for global population. Unmet need for family planning is crucial to securing the well-being and sovereignty of women. The unprecedented population growth has also been considered as the primary drivers of various ecological, societal threats and mass species extinction (1). Hence, the concept of contraception has a paramount impact in regulating overpopulation (2). Males are typically restricted to condoms and vasectomy; consequently, females are often burdened with the responsibility of population regulation (3). The approaches for females are usually achieved through barrier methods, hormonal, non-hormonal, chemicals such as quinacrine, intrauterine devices, tubectomy, and transcervical sterilization. In India, sterilization methods dominate over all other contraceptives in females. Adolescents have their reproductive careers ahead of them, they also require to postpone or space pregnancies, which requires reversible and non-invasive methods. Female sterilization or tubectomy is meant to be permanently associated with health risk without preventing sexually transmitted diseases. It is an irreversible method, which can be reversed by canalization, but this is not always successful. If pregnancy occurs after the procedure, there is an increased risk for an ectopic pregnancy (4). Thus, limitations are still associated with tubectomy, and therefore, further research on efficacy and reversibility is needed to achieve better contraceptive modalities. So, in the light of extensive use of tubectomy along with drawbacks, there is still a lapse in an ideally accepted contraceptive method in terms of efficacy, safety, and reversibility for females.

A polymer-based contraceptive, Reversible Inhibition of Sperm Under Guidance (RISUGⓇ) is currently in advanced phase III clinical trials, which could be an effective male contraceptive with the potential of reversibility (5, 6). The contraceptive activity of RISUGⓇ as a potential molecule along with safety, efficacy, non-toxicity, and reversibility has been successfully confirmed in animal models, *viz.*, rats, rabbits, and langur monkeys (5-12). Non-invasive approaches have been applied successfully in monkeys for the vas occlusion reversal (7, 12). Reversal with DMSO and/or NaHCO${}_{3}$ has also been studied in rats and rabbits proving its feasibility and safety up to F${}_{1}$ generation with restoration of complete fertility (6, 9-12). RISUGⓇ exerts contraceptive effects by occlusion at delivery site, lowers pH, charges disturbance in gamete and excessive generation of reactive oxygen species. Its in vitro ovicidal properties also suggest RISUGⓇ as a potential female fertility-regulating agent (13, 14).

Thus, this experimental study has been designed with an objective to develop a concept of non-invasive, transcervical, single intervention, and reversible female contraceptive using RISUGⓇ as a fallopian tube implant. Therefore, in the present investigation, the contraceptive efficacy of RISUGⓇ-induced tubal occlusion and functional reversal with dimethyl sulphoxide (DMSO) and NaHCO${}_{3}$ was carried out following a short-term reversal in female albino rats.

## 2. Materials and Methods

### Drug

RISUGⓇ, a copolymer of styrene and maleic anhydride (1:1 ratio), was prepared through cobalt 60 gamma irradiation of styrene and maleic anhydride monomer in the presence of nitrogen in ethyl acetate at a dose of 0.2-0.24 mega rad for every 40 g of polymer at a dose rate of 30-40 rad/sec. After that, it was precipitated, dried, and stored in a vacuum desiccator, dissolved in DMSO solvent (15). The drug was provided by Prof. S. K. Guha, School of Medical Science and Technology, Indian Institute of Technology, New Delhi, India.

### Animals

Adult Wistar albino female rats *(Rattus norvegicus)*, weighing 150-155 gr, 3-4 months old were used for the study. The animals were hygienically maintained at the Departmental Experimental Animal facility, with well-regulated photoperiod (12 hr light: 12 hr dark), in individual polypropylene cages (size 43 × 27 × 15 cm). The temperature in animal house was adjusted at 23 ± 2°C, and the relative humidity ranged between 32 and 70%. Animals were fed twice a day with rat pellet diet (Ashirwad Industries Limited, Chandigarh, India) and safe drinking water *ad libitum*.

### Experimental design

A total of 60 animals were included in this experimental study. Group I (Sham-operated control) contained 30 animals (Sub-groups Ia, Ib, and Ic (n = 10/each)), while groups II, III, and IV contained 10 animals each. Animals in the sub-groups served as parallel controls for groups II-IV to determine the fertility among the experimental groups. In group I, the animals were subjected to bilateral tubal occlusion, but no drug was injected and served as control; group II: the animals were subjected to tubal occlusion with 5-7 µL of RISUGⓇ, bilaterally, and euthanized after 90 days; group III: the animals were subjected to tubal occlusion reversal with DMSO after 90 days of occlusion; group IV: the animals were subjected to tubal occlusion reversal with 5% NaHCO${}_{3}$ after 90 days of occlusion. Same animals were repeated at every mating schedule. The repeated mating ensured the fertility at given interval. Following the last mating schedule, animals were sacrificed.

### Methodology

#### Tubal occlusion

Animals of groups II-IV were subjected to bilateral fallopian tube occlusion, after an overnight fasting, under sodium thiopentone anesthesia (20 mg/kg body weight, intraperitoneal; Thiosol Sodium: Neon Laboratories Ltd., Mumbai, India). Skin was disinfected and lateral incision was made to locate the uterus. Using the microsyringe fitted with blunt needle (26 G), a 5-7 µL of therapeutic standardized dose of RISUGⓇ was injected into each fallopian tube to block the median to distal segment (antegrade). RISUGⓇ polymerization was catalyzed by application of normal saline and solidified on contact with luminal fluid. The tube was placed in its original position and incision was closed by catgut suture in the inner and by silken suture in the outer skin. Postoperative care was acquired through antibiotic Ceftriaxone injection 0.025 mg/ml i.p. (Cefoat, A to Z Pharmaceuticals Ltd., Gujarat, India), anti-inflammatory Meloxicam 0.025 ml i.p. (Melonex, Intas Pharmaceuticals Ltd., Gujarat, India), and topical HealexPlus Spray (Shreya Life Sciences Pvt. Ltd., Mumbai, India). Until healing, the animals were supervised and provided proper bedding, feed, and water. The fallopian tubes of group I animals (sham-operated control) were exposed in a similar way, but no drug was injected.

#### Tubal occlusion reversal

The reversal was performed following 90 days of tubal occlusion. The tubes were exposed in a similar way as that of tubal occlusion and injected bilaterally with 250-500 µL of DMSO in group III and 500-700 µL of 5% NaHCO${}_{3}$ in group IV animals to liquefy RISUGⓇ (11). The dissolution of RISUGⓇ was confirmed by free flow of the DMSO and 5% NaHCO${}_{3}$ through the entire length of tube and DMSO and 5% NaHCO${}_{3}$ swill out due to the positive pressure emanating from the tube through the vagina via uterus. The tube was returned to original position and postoperative care was provided as described previously.

### Parameters

#### Monitoring of estrous cycle

Estrous cycle was screened by taking vaginal swabs (16). Vaginal secretion was collected with the sterile glass dropper filled with 10-20μL of normal saline (0.9% NaCl) by inserting the tip smoothly into the vagina and gently flushing the saline in and drawing it back into the glass dropper. The vaginal fluid was uniformly spread on the marked slides and observed under the light microscope (Model: DM1000, Leica Wetziar, Germany).

#### Fertility test

Periodical fertility tests were conducted in sham operation, tubal occlusion, and reversal groups as per following schedule, *viz.,* 30, 60, and 90 days of tubal occlusion and 45, 105, 150, and 195 days of tubal occlusion reversal by cohabitating the experimental females with the proven fertile male rats at 1:1 ratio. Mating success was confirmed by the appearance of vaginal plug and presence of spermatozoa in the vaginal smear examined under light microscope (Model: DM1000, Leica Wetziar, Germany). The number of females that delivered and the litter size was recorded for calculating fertility.

#### Histology of fallopian tube

Fallopian tube from all animals was removed surgically at scheduled sacrification and fixed overnight in 4% paraformaldehyde, dehydrated in ethanol, cleared in xylene, and embedded in paraffin wax. Next, 5-µm thin sections were cut, fixed, spreaded on Mayer's albumin-coated slides and processed for hematoxylin and eosin staining for light microscopic observations (Model: DM1000, Leica Wetziar, Germany).

### Ethical considerations

The experimental protocol was approved by the Institutional Animal Ethics Committee (IAEC) under certificate no. UDZ/IAEC/I/02, dated 03.07.2015, Department of Zoology, University of Rajasthan, Jaipur, India. Animals were maintained under perfect veterinary supervision, and the guidelines of “Committee for the Purpose of Control and Supervision of Experiments on Animals” (17) were strictly followed.

### Statistical analysis

Data are represented as mean ± standard deviation (SD). One-way analysis of variance (ANOVA) test followed by post-hoc (Duncan's new multiple range test) procedure was applied for statistical comparisons, wherever applicable. Data were analyzed using the Holm-Sidak multiple comparison test to detect the inter-group difference using the statistical software SPSS, version 10.0 (SPSS Inc., Chicago, IL, USA). P < 0.05 was considered as significant.

## 3. Results

### Characteristics of estrous cycle

**Figure 1:**
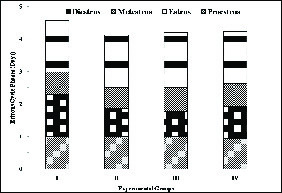
Estrous cycle phases (days) of female rats following sham operation, tubal occlusion with RISUGⓇ, and its reversal with DMSO and NaHCO${}_{3}$. Group I (control group) was further divided into three sub-divisions for comparing the fertility with the experimental groups (II, III, and IV). Thus, 10 animals were chosen randomly for comparing estrous phases with other experimental groups (II, III, and IV).

The estrous cycle phases, that is, proestrus, estrus, metestrus, and diestrus were found to be comparable to the control. Thus, the estrous cycle length was normal, 4-5 days long in the animals of groups II-IV when compared with group I animals (Figure 1).

### Fertility test

The presence of spermatozoa in the vaginal smear along with vaginal plug confirmed successful mating in all groups. Animals of group I showed 100% fertility during all mating schedules of pre-injection, tubal occlusion, and reversal periods. Animals of group II- IV indicated positive mating, but 0% fertility was evident following 30, 60, and 90 days of tubal occlusion. In groups III and IV, animals exhibited 0% fertility following tubal occlusion and gradual increase in fertility following reversal was observed. However, group III animals exhibited gradual increase in fertility (50% at 45 days, 80% at 105 days, 100% at 150 days, and 100% at 195 days) following tubal occlusion reversal with DMSO. Group IV animals indicated a gradual enhancement in fertility (70% at 45 days, 70% at 105 days, 100% at 150 days, and 100% at 195 days) following tubal occlusion reversal with NaHCO${}_{3}$ during all mating schedules, respectively. No remarkable changes were observed in the mean litter size in all experimental groups as compared with group I (Table I).

### Histology of fallopian tube

**Figure 2:**
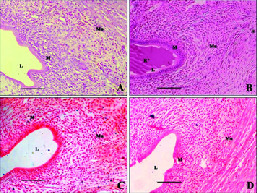
Histology of rat fallopian tube. (A) Group I (sham-operated control) rat showing normal histological features, illustrating a well-defined inner mucosa, muscularis, and outer serosa. The tubal isthmus appears thick-walled, more muscular, with narrow lumen and short mucosal folds. (B) Group II (tubal occlusion with RISUGⓇ for 90 days) rat showing shedded epithelium with blunt and low villi and eosinated implant in luminal epithelial cells of the isthmus region. (C) Group III (tubal occlusion with RISUGⓇ for 90 days and its reversal with DMSO) rat showing unaltered features as compared to control. (D) Group IV (tubal occlusion with RISUGⓇ for 90 days and its reversal with NaHCO${}_{3}$) rat showing unaltered features as compared to control (HE x 200). Scale Bar = 50 µm. S: Serosa; Mu:Muscularis; M: Mucosa; L: Lumen; R*: RISUG implanted.

The fallopian tubes of group I animals revealed normal histological features, illustrating a definite inner mucosa, circular and longitudinal muscularis, and outer serosa. The tubal isthmus was noticed with thick wall, more muscular with narrow lumen and short mucosal folds (Figure 2A). The fallopian tubes of group II animals revealed distorted cellular configuration along with disordered epithelial cells at certain places in the implanted region; nonetheless, normal histological architecture of mucosa, muscularis, and serosa were observed. In the implanted isthmus, mucosal folds were irregular with disturbed structural pattern. The accumulation of shedded and detached epithelial tissues from mucosal folds and the isthmic lumen filled with eosinated RISUGⓇ implant were observed (Figure 2B). The fallopian tubes of reversal groups (III and IV) animals revealed complete regeneration of the epithelial linings when compared with the controls (Figures 2C and 2D).

**Table 1:** Fertility record of female rats following sham operation, tubal occlusion with RISUGⓇ and its reversal with DMSO and NaHCO${}_{3}$


<**Mating schedule groups**		**Pre-injection**	**Tubal occlusion**			**Tubal occlusion reversal**			
	**0 day**	**30 days**	**60 days**	**90 days**	**45 days**	**105 days**	**150 days**	**195 days**
**No. of females delivered/mated**									
	**Ia**	10/10	10/10	10/10	10/10	**-**	**-**	**-**	**-**
	**II**	10/10	0/10	0/10	0/10	**-**	**-**	**-**	**-**
	**Ib**	10/10	10/10	10/10	10/10	10/10	10/10	10/10	10/10
	**III**	10/10	0/10	0/10	0/10	05/10	08/10	10/10	10/10
	**Ic**	10/10	10/10	10/10	10/10	10/10	10/10	10/10	10/10
	**IV**	10/10	0/10	0/10	0/10	07/10	07/10	10/10	10/10
**Litter size**									
	**Ia**	5.00 ± 0.89	5.10 ± 0.83	4.20 ± 0.87	4.40 ± 1.11	**-**	**-**	**-**	**-**
	**II**	4.50 ± 0.50	0	0	0	**-**	**-**	**-**	**-**
	**Ib**	4.80 ± 0.75	4.90 ± 0.83	4.80 ± 1.08	5.00 ± 1.18	4.10 ± 0.70	4.20 ± 1.08	5.10 ± 0.70	4.50 ± 1.03
	**III**	4.70 ± 1.00	0	0	0	5.00 ± 0.89	4.90 ± 0.83	4.40 ± 0.80	4.20 ± 1.33
	**Ic**	4.50 ± 0.81	4.60 ± 0.92	5.00 ± 0.63	4.30 ± 0.90	4.70 ± 1.10	4.80 ± 0.87	5.10 ± 0.94	4.30 ± 0.78
	**IV**	4.40 ± 0.49	0	0	0	4.80 ± 0.83	4.20 ± 0.99	4.10 ± 0.70	4.60 ± 1.02
**% Fertility**									
	**Ia**	100	100	100	100	**-**	**-**	**-**	**-**
	**II**	100	0	0	0	**-**	**-**	**-**	**-**
	**Ib**	100	100	100	100	100	100	100	100
	**III**	100	0	0	0	50	80	100	100
	**Ic**	100	100	100	100	100	100	100	100
	**IV**	100	0	0	0	70	70	100	100
All data are expressed as Mean±SD. Group Ia, Ib, Ic: sham-operated control; group II: tubal occlusion with RISUGⓇ for 90 days; group III: tubal occlusion with RISUGⓇ for 90 days and reversal with DMSO; group IV: tubal occlusion with RISUGⓇ for 90 days and reversal with 5% NaHCO${}_{3}$									

## 4. Discussion

The results of the present investigation demonstrate that the RISUGⓇ-induced complete sterility following 90 days of tubal occlusion and reversibility with both DMSO and NaHCO${}_{3}$ without any changes in estrous cyclicity. Histology of fallopian tube revealed distortion of epithelial cell configuration in the implanted site in tubal occluded animals and complete regeneration of the epithelial linings following reversal. The alarming population growth demands better contraceptive modalities to expand the contraceptive selections for both sexes. Tubectomy is an extensively exercised surgical contraceptive, yet post-tubectomy pregnancies and abnormalities have been reported (18). The sale and use of EssureⓇ, the first US Food and Drug Administration (FDA)-approved standard transcervical procedure was restricted due to Essure problems (19). The second FDA-approved AdianaⓇ has also been associated with menstruation cramping, intractable pain, tubal perforations, and ectopic pregnancies (4, 20-22). Quinacrine sterilization is less acceptable as it was proved as a mutagen (23, 24). Recently discovered polidocanol foam (PF) also resulted in nonsurgical female contraception through tubal occlusion via multiple treatments in baboons (25) and rhesus (26). An *ex vivo* macaque fallopian tube has also been employed for assessing the PF outcomes (27). Conversely, further research has been warranted to induce permanent tubal occlusion with a single transcervical intervention and successful reversibility. These reports exceedingly warrant substitute contraceptives with minimal invasive approach.

In the present study, the RISUGⓇ has been investigated as a single intervention approach with a possibility of reversal. The drug was injected through micro-syringe via distal section (infundibulum with fimbriae) occluding the tubes and consequently hindering capacitation. The fimbriae have crucial role in sweeping the oocyte into the fallopian tubes for fertilization. If they no longer function then the oocyte never makes it to its destination for fertilization (28). RISUGⓇ implants in fallopian tubes appear to block the tubes on solidification, alter the pH and osmolarity, thus, generating unfavorable conditions for fertilization. As reproductive tract, pH is the chief determinant in the sperm-ova interaction; an elevated pH can adversely affect cervical mucus and sperm cell motility leading to infertility (29). The implants may adversely affect the ova morphology on interaction, making it non-viable by generating reactive oxygen species. In order to achieve reversal, the RISUGⓇ blockage was flushed out favorably through DMSO and NaHCO${}_{3}$, normalizing the fallopian tubes as well as oocyte movement, subsequently resuming fertility with normal F${}_{1}$ progeny. Simultaneously, various parameters proved safety, efficacy, and reversibility of the procedure (9, 10, 13). Further investigations in a suitable non-human primate model will open avenues for clinical trials to adjudge suitability of RISUGⓇ as a non-invasive female contraceptive.

## 5. Conclusion

RISUGⓇ proved to be suitable for single intervention, intratubular, reversible contraception in female rats.

##  Conflict of Interest

The authors declare that there is no conflict of interest.
